# Harnessing tumor-associated macrophages as aids for cancer immunotherapy

**DOI:** 10.1186/s12943-019-1102-3

**Published:** 2019-12-05

**Authors:** Xiaolei Li, Rui Liu, Xiao Su, Yongsha Pan, Xiaofeng Han, Changshun Shao, Yufang Shi

**Affiliations:** 1 The First Affiliated Hospital of Soochow University, State Key Laboratory of Radiation Medicine and Protection, Institutes for Translational Medicine, Soochow University Medical College, 199 Renai Road, Suzhou, 215123 Jiangsu China; 2 Center of Translational Medicine, Affiliated Wuxi No.2 People’s Hospital of Nanjing Medical University, 68 Zhongshan Road, Wuxi, 214002 Jiangsu China

**Keywords:** Tumor immunology, Immunotherapy, Immune checkpoint blockade, Tumor-associated macrophage, Combination therapy

## Abstract

Cancer immunotherapies that engage immune cells to fight against tumors are proving to be powerful weapons in combating cancer and are becoming increasingly utilized in the clinics. However, for the majority of patients with solid tumors, little or no progress has been seen, presumably due to lack of adequate approaches that can reprogram the local immunosuppressive tumor milieu and thus reinvigorate antitumor immunity. Tumor-associated macrophages (TAMs), which abundantly infiltrate most solid tumors, could contribute to tumor progression by stimulating proliferation, angiogenesis, metastasis, and by providing a barrier against antitumor immunity. Initial TAMs-targeting strategies have shown efficacy across therapeutic modalities and tumor types in both preclinical and clinical studies. TAMs-targeted therapeutic approaches can be roughly divided into those that deplete TAMs and those that modulate TAMs activities. We here reviewed the mechanisms by which macrophages become immunosuppressive and compromise antitumor immunity. TAMs-focused therapeutic strategies are also summarized.

## Background

The advent of cancer immunotherapies, including checkpoint blockade-based immunotherapy and adoptive cell therapy, has provided new options and powerful weapons to combat cancer [1]. The development of now immunotherapies has revolutionized the field of oncology, leading to successful clinical trials for multiple cancer types. However, for the majority of patients with solid tumors, little or no progress has been seen [2]. When tumor develops from neoplastic tissue to clinically detectable tumors, malignant cells acquire different mechanisms that mimic peripheral immune tolerance to evade the immune surveillance and to avoid tumoricidal attack. Unfortunately, the efficacy of the current immunotherapies is limited by the multifaceted immunosuppressive signals within the tumor microenvironment (TME) [3, 4]. Thus, new strategies that can efficiently reprogram the various immunosuppressive cells in the TME and further elicit antitumor immunity are urgently needed.

Solid tumor evolves as a complex ecosystem involving active interactions between tumor cells and stromal cells [5]. Inflammatory cells make up a significant proportion of the overall tumor mass, and among them, macrophages, called tumor-associated macrophages (TAMs), represent one of the most abundant stromal components in the TME and are therefore conspicuous stromal targets in many, if not all, solid tumors [6, 7]. In fact, TAMs have a dominant role as orchestrators of cancer-related inflammation that is now recognized to be an integral factor that accelerates tumor progression and limits the response to antitumor immunity [8–10]. Mechanistically, TAMs could build up and remodel the extracellular matrix structure, which enables the tumor cells to invade through the TME and interact with tumor cells or other stromal cells via the secretion of growth factor, cytokines and chemokines [8, 10].

**Fig. 1:**
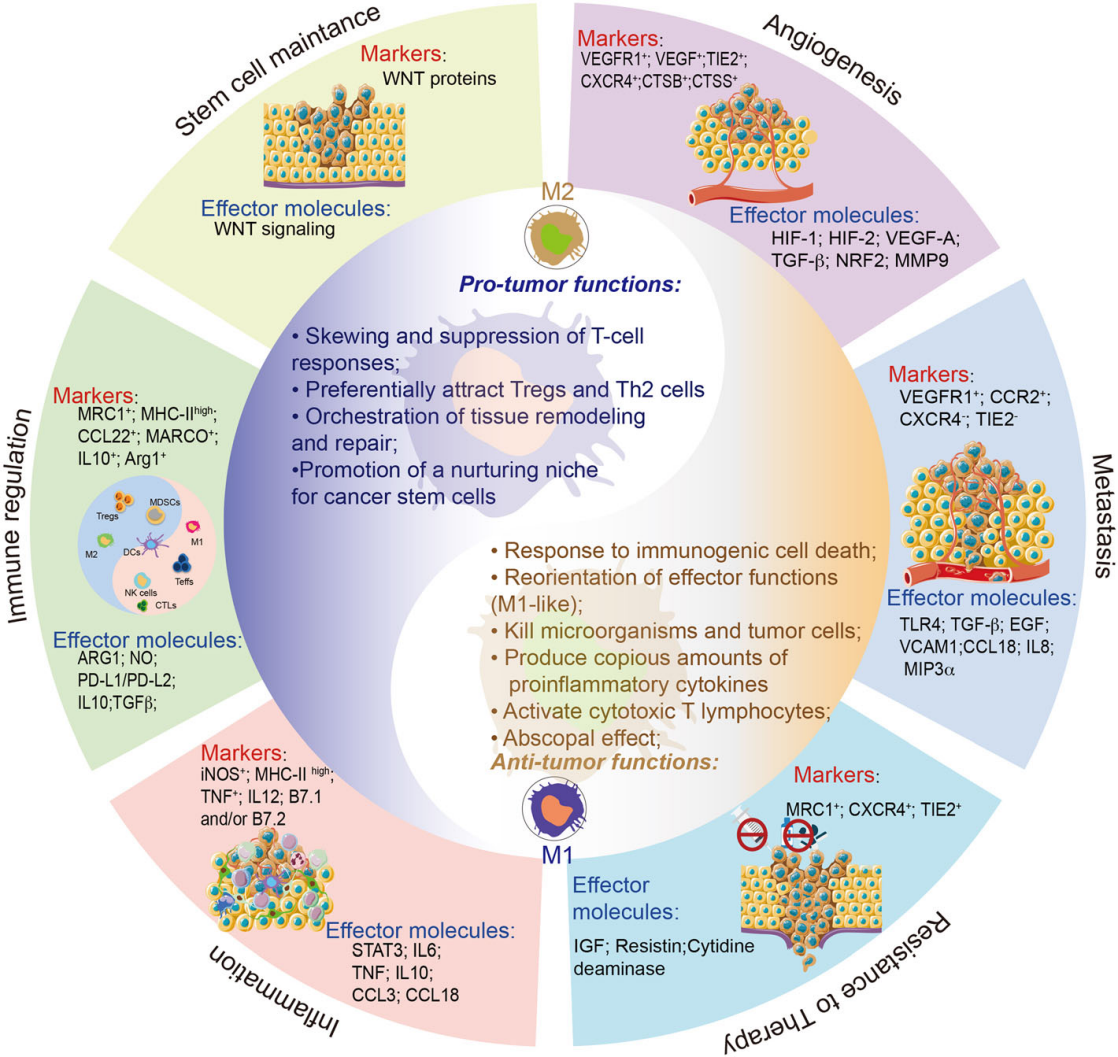
The yin and yang of TAMs in response to cancer immunotherapy. Macrophages have bimodal, yin and yang roles in orchestrating immune responses, and can either hamper (left-hand side), or foster (right-hand side) the effectiveness of cancer immunotherapy strategies. The two macrophage subtypes are defined as M1 and M2. M1 macrophages, also known as ‘killer’ macrophages, were previously referred to as classically activated macrophages; In contrast, the M2 macrophages, also known as “repair” macrophage, are referred to as the alternatively activated macrophages. The M2 macrophages contribute to constructive processes, including wound healing and tissue repair. In addition, the M2 macrophage prevents immune surveillance system by producing immuno-suppressing cytokines. This differential polarization is under the control of many stimuli that alters the differentiated state of the macrophages. At present, TAMs are believed to promote cancer initiation and malignant progression by stimulating tumor-associated angiogenesis, promoting tumor cell metastasis, invasion and intravasation, chemotherapeutic resistance as well as suppressing the response to antitumor immunity

Macrophages can be either embryonically seeded in tissues, where they are maintained through self-renewal, or derived from monocyte precursors, which infiltrate tissues and differentiate in response to their microenvironment [11]. TAMs, at least in mouse models, are thought to predominantly derive from circulating bone marrow monocytes driven by inflammatory cues from tumor cells in the primary and metastatic tumor, where they differentiate into TAMs and facilitate tumor progression [12, 13]. However, in tumors such as gliomas and pancreatic cancer, TAMs can also be derived from myeloid progenitors developed in the yolk sac at the embryonic stage [14–16]. Nevertheless, in either case, signals originating from tumor cells, lymphocytes, and stromal cells influence TAMs function and diversity. Owing to their preponderance, TAMs are relatively easy to isolate and thus have been extensively studied. The classification of activated macrophages activation as either classical (M1; interferon (IFN)-γ/lipopolysaccharide (LPS)-dependent) or alternative (M2; interleukin-4 (IL-4)/IL-13/IL-10 dependent) has provided a necessary framework for the understanding of TAMs polarization [17, 18]. However, accumulating evidence suggests that this is an oversimplification and their complexity would be better described as a dynamic spectrum of phenotypes. As a reflection of the great diversity and plasticity of macrophages, the phenotype and composition of TAMs vary between tumor types, which have been reviewed in detail elsewhere [19, 20]. TAMs could exert pleiotropic protumor activities, while they also contribute to antitumor immunity, depending on ontogeny, tissue-specific regulation, and tumor stage (Fig. 1). Accordingly, evidence indicates that, in nascent tumors, TAMs display an M1-like phenotype and can eliminate some immunogenic tumor cells. Subsequently, tumor progression is associated with skewing and subversion of macrophage function by the cues within TME that could elicit an M2-like polarization of TAMs that is pro-tumorigenic [10, 21]. Till date, TAMs are believed to promote cancer initiation and malignant progression by stimulating tumor-associated angiogenesis, promoting tumor cell metastasis, invasion and intravasation, chemotherapeutic resistance as well as suppressing the response to antitumor immunity [22, 23].

Considering the multifaceted roles of TAMs in tumor development, selective targeting of the immunosuppressive TAMs in the TME in ways that could synergize with the current cancer immunotherapy thus presents an attractive strategy [10, 24]. Targeting TAMs, which function upstream of T cell responses, may complement the current cancer immunotherapies that purely modulate T cells, such as adoptive cell therapy or immune checkpoint blockades [25]. Novel strategies aimed at modulating the TME have resulted in various TAMs-directed approaches, ranging from macrophage depletion to macrophage repolarization. In this review, we discuss the underlying mechanisms by which TAMs suppress antitumor immune responses. We further suggest that ablation or re-education of macrophages within the TME may become an important prong of combination therapies designed to combat cancer. We also highlight the current findings about the immunomodulatory effects of TAMs on cancer immunotherapy, especially checkpoint blockade-based immunotherapy.

### TAMs as enabler of the tumor immunosuppressive milieu

Solid tumors are complex ecosystems, defined by the interplay of a large number of cellular and soluble components [4]. Macrophages are one of the major components of the leukocyte infiltrate in the TME and play a prominent role in the evasion from established immune surveillance, and their accumulation has been generally associated with poor prognosis in solid tumors [8, 11, 23].

Numerous studies have demonstrated that TAMs could suppress naïve T cell proliferation in vitro, suggesting that macrophages can directly suppress T cell function [26, 27]. TAMs can inhibit cytotoxic T cell responses by several means: 1) depletion of metabolites essential for T cell proliferation; 2) inhibition of T cell functions by producing anti-inflammatory cytokines; 3) activation of T cell checkpoint blockade via engaging inhibitory receptors.

Arginine metabolism has crucial roles in T cell activation and in the modulation of immune responses [28]. TAMs can inhibit T cell activity by the depletion of L-arginine in the TME by secreting Arginase 1 (ARG 1), which is characteristically expressed in M2-like murine macrophages and in many TAM populations. L-arginine, which is required for T cells function, could be metabolized to L-ornithine, as well as other anti-inflammatory products, such as urea, by ARG 1 in the TME. L-arginine depletion results in the failure to re-express CD3 ζ-chain in the T cell receptor complex and inability to respond to tumor antigen, thus suppressing effector T cell activation [28]. L-arginine is also the substrate of the inducible nitric oxide synthase (iNOS). Intriguingly, both of the L-arginine catabolic pathways, mediated by ARG 1 and iNOS respectively, lead to T cell suppression, though by different mechanisms [29]. However, iNOS expression by myeloid cells, leading to enhanced recruitment of adoptively transferred T cells, has also been implicated in promoting a T cell response [30]. Thus, the impact of iNOS expression by macrophages may be highly context-dependent.

Under the influence of tumor-derived factors, TAMs could also secrete an array of cytokines, including IL-10, transforming growth factor-β (TGF-β) and prostaglandin-E2 (PGE2), that further inhibit T cell-mediated immune response to establish a self-propagating immunosuppressive TME [8, 23, 27]. Despite being initially identified as a growth factor and then found to be a tumor suppressor, TGF-β plays an important role in supporting and regulating tumor development and metastasis, as well as tumor-directed immune responses. Particularly, TGF-β exerts a multifunctional effect on adaptive immune cells, regulating both effector and regulatory T cells (Tregs) and cytotoxic T lymphocytes (CTLs) and supporting generation of immunosuppressive cells (reviewed in [31]). TGF-β in combination with IL-2 enforces a suppressor phenotype in naïve CD4^+^ T cells ex vivo by triggering expression of forkhead box P3 (FOXP3), the master transcription factor of the Tregs [32]. Macrophages in the intestinal immune system have been shown to induce Tregs by the secretion of IL-10 and TGF-β [33]. IL-10 could also suppress the function of multiple immune cells, thus playing a crucial role in dampening antitumor immunity while supporting tumorigenesis. In fact, IL-10, as a pleiotropic cytokine, exerts diverse effects on most immune cells, with the ability to inhibit activation and effector function of T cells, as well as monocytes and macrophages (reviewed in [31]). Noticeably, IL-10 expressing anti-inflammatory macrophages are responsible for induction of Tregs [34]. IL-10 production by TAMs could blunt antitumor responses by inhibiting the functions of antigen-presenting cells (APCs) and subsequently blocking T cell effector functions, such as cytotoxicity (see review in [35]). Studies in mouse tumor models have shown that IL-10 can suppress the maturation of intratumoral dendritic cells (DCs) and their production of IL-12, and thereby limits cytotoxic T cell responses during chemotherapy [27]. In addition, IL-10 can also directly act on CD8^+^ T cells to exert specific inhibitory effects by increasing N-glycan branching, thereby reducing co-localization of CD8 protein with T cell receptor (TCR) [36]. Galectin 3 binding glycoproteins on the surface of T cells plays a central role in mediating the IL-10 induced reduction in CD8^+^ T cell antigen sensitivity, and TCR-CD8 co-localization and IFN-γ expression by CD8^+^ T cells from human ovarian ascites can be restored by N-acetyllactosamine (LacNAc) treatment, a galectin disaccharide ligand to interfere with galectin 3 binding glycoproteins [36]. Since macrophages can be an important source of galectin 3 in inflamed tissues [37], TAMs may even regulate multiple immune suppressive functions of IL-10.

TAMs-induced immune suppression is also mediated by the expression of inhibitory receptors, including classical and nonclassical major histocompatibility complex class I (MHC-I) molecules, which are normally associated with the presentation of antigens to T cells. Macrophages can express human leukocyte antigen (HLA) molecules, such as HLA-C (classical), HLA-E, and HLA-G (nonclassical), which could inhibit the activation of NK cells and a subsets of activated T cells upon their ligation to CD94 (also known as NKG2) and leukocyte immunoglobulin-like receptor subfamily B member 1 (LIR1, also called as ILT2), respectively (also reviewed in [8]). In addition to these MHC molecules, TAMs also express PD-L1, PD-L2, CD80 and CD86, the ligands to inhibitory receptors programmed cell death protein 1 (PD-1) and cytotoxic T-lymphocyte antigen 4 (CTLA-4), which are normally upregulated in activated immune effector cells, including T cells, B cells, and NK T cells, as a part of a safety mechanism that controls the intensity of the immune response and as well as a pathway to inflammation resolution [2, 10, 11, 25]. Taken together, TAMs may directly inhibit T cell functions via these immune checkpoint ligands, while also secreting IL-10 and TGF-β to elicit immunosuppressive functions.

Other immune checkpoint ligands expressed by TAMs potentially also have direct suppressive effects on tumor-infiltrating T cells, such as B7-H4 (aka B7x, B7S1 or VTCN1) and V-domain Ig- containing suppressor of T cell activation (VISTA, aka PD-1H, DD1α), which might confer macrophages immunosuppressive capacity [38–40]. B7-H4, a relatively new member of the B7 superfamily that was identified in 2003, shares approximately 25% amino acid homology with other B7 family members in the extracellular portion [41–43]. B7-H4 expressed on TAMs was implicated in suppression of T cell activation [39, 44]. Although the receptor for B7-H4 remains unknown, B7-H4-expressing cells and its-immunoglobulin fusion proteins could act as negative regulators of T cell responses by inhibiting T cell proliferation, cell cycle progression, and cytokine production [41, 44]. Of the utmost importance, the surface B7-H4 on TAMs, but not the intracellular B7-H4 in primary ovarian tumor cells, was shown to suppress tumor-associated antigen-specific T cell immunity [39]. Moreover, inhibition of B7-H4 restores the T cell stimulating function of TAMs that contributes to tumor regression, and also suppresses the growth of subcutaneously implanted tumors in mice by reducing CD8^+^ T cell exhaustion [39]. Noticeably, B7-H4 expression on TAMs was found to positively correlate with the clinical stage of patients with tumors [45]. However, the specific role that B7-H4 plays in tumor immunity may be context dependent [46, 47]. The dichotomy in the function of B7-H4 may reflect the specifics of the tumor model used, especially as B7-H4 expression could be regulated by cytokines commonly present in the TME, such as IL-6, IL-10 and IFN-γ [46]. VISTA is another recently-discovered immune regulator protein with a similar structure to the B7 Ig superfamily, expressed in lymphoid organs and on myeloid cells [40, 48]. VISTA functions as an immunosuppressive molecule both as a ligand on APCs and as a receptor on T cells, resulting in stunted T cell cytokine production, blockade of T cell proliferation, and induction of Foxp3 expression, and thus sustaining the pool of Tregs [48]. The expression of VISTA on tumor-infiltrating T cells and TAMs was elevated in prostate cancer and melanoma patients following ipilimumab treatment, with a greater proportion of macrophages with VISTA expression being of the immunosuppressive M2 phenotype, suggesting that VISTA may represent a compensatory resistance mechanism [38].

Other possible mechanisms by which macrophages could inhibit effector T cell function indirectly could be through the production of chemokines that recruit natural Tregs to the TME, and through the secretion of an array of cytokines that induce the Tregs and sustain their survival. TAMs-released chemokines, including C-C chemokine ligand (CCL)-2, CCL-3, CCL-4, CCL-5, CCL-20, and CCL-22, further contribute to the recruitment of Tregs in the TME [25, 49]. In addition to their function as a recruiter, macrophages could be involved in restricting the intratumoral localization of T cells. The mechanisms by which TAMs prevent CD8^+^ T cells from reaching tumor cells are still not known. Fibrosis would be another underlying mechanism by which macrophages could shield TME from T cell infiltration. Macrophages actively participate in tissue remodeling through interaction with fibroblasts, whereby collagen is synthesized and secreted, and organized into bundles in construction of the tissue in developing mammary gland [50, 51]. Additionally, TAMs were shown to produce and remodel collagenous extracellular matrix [52]. Intriguingly, TAMs can produce granulin to induce fibrosis in tumor stroma, thus excluding T cells from tumor mass [53, 54]. TAMs are the main TGF-β producers in many tumor types. TGF-β can affect both the adaptive and innate immune systems [55, 56], and also contributes to the evasion of immune surveillance [57, 58]. TGF-β also affects TAMs by increasing their immunosuppressive activity. Thus, it is possible that macrophages could act to exclude T cells from tumors through activation of TGFβ. Although many TGFβ inhibitors have been investigated in both preclinical and clinical studies, TGFβ pleiotropic activity, the biological differences between TGFβ1, TGFβ2 and TGFβ3, and the multiple regulation of TGFβ make it a challenging target.

Taken together, TAMs act as central drivers of the immunosuppressive TME through their expression of cell surface receptors, secreted cytokines, chemokines, and enzymes that regulate the recruitment and the function of multiple immune subtypes.

### Emerging strategies for targeting TAMs

**Fig. 2:**
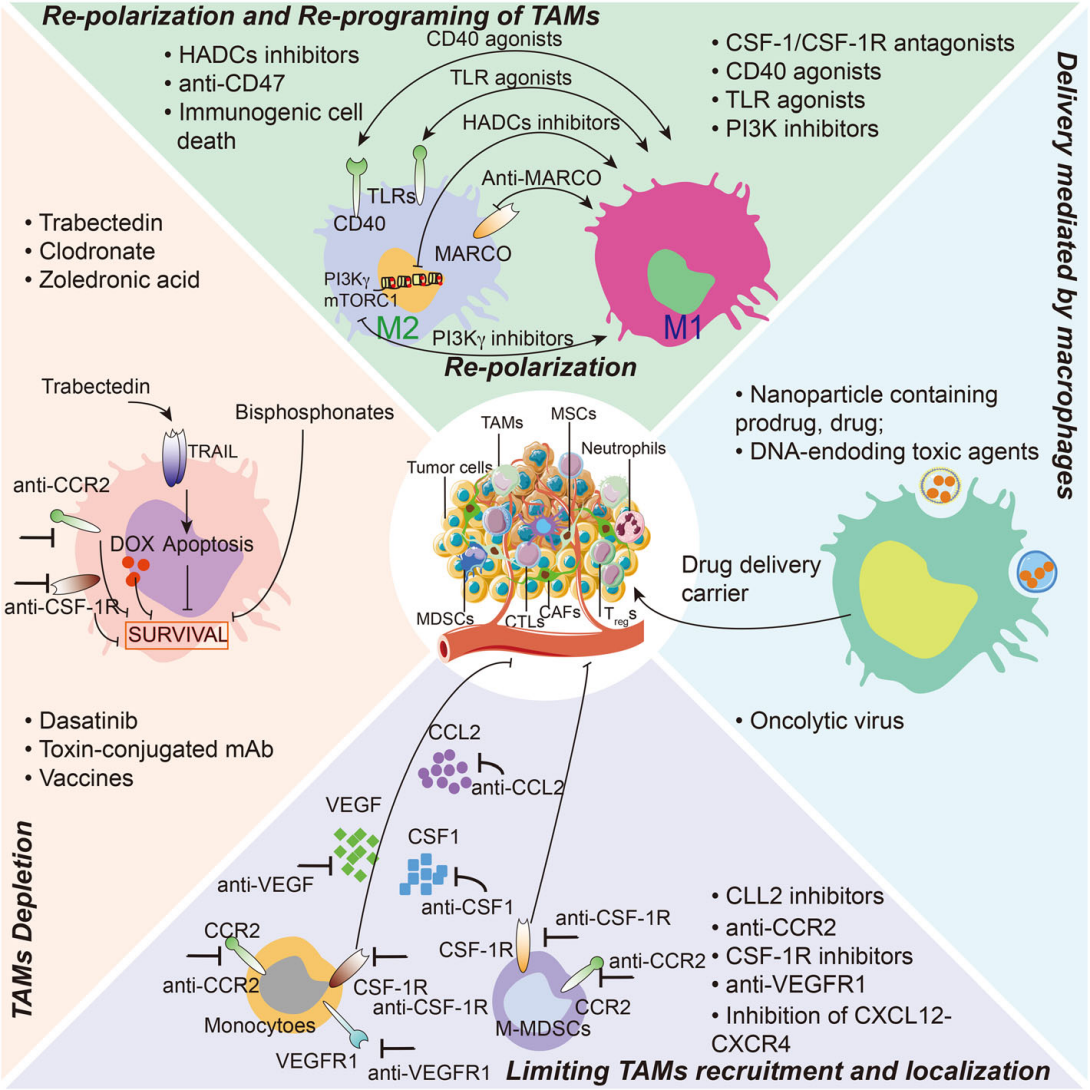
Principal strategies for TAMs-directed antitumor therapy. Four general approaches that target or utilize TAMs as cancer therapy are discussed clockwise, starting at the below. The strategies fall into four main groups: i) inhibition of TAMs recruitment to the tumor, ii) direct killing of TAMs, iii) re-education of TAMs from their M2-like protumoral phenotype into a M1-like antitumoral phenotype. iv) TAMs-mediated delivery of therapeutic cargoes. CAFs, cancer-associated fibroblasts; CTLs, cytotoxic T lymphocytes; CSF1, colony-stimulating factor 1; CSF1R, CSF1 receptor; PI3Kγ, phosphoinositide 3-kinase-γ; HDAC, histone deacetylase; MSCs, mesenchymal stem/stromal cells; MARCO, macrophage receptor with collagenous structure; MDSCs, myeloid-derived suppressor cells; Tregs, regulatory T cells; TLRs, toll-like receptors; VEGF, vascular endothelial growth factor; VEGFR, VEGF receptor

The protumoral properties of TAMs during tumor progression make them tempting therapeutic targets for cancer treatment [8, 11, 24, 25]. Current strategies are designed to diminish TAMs directly or to polarize TAMs toward a tumoricidal phenotype. These strategies can be further divided into four main groups (Fig. 2): 1) inhibition of macrophages recruitment to the tumor, 2) direct killing of TAMs, 3) conversion of TAMs from their M2-like protumoral phenotype to a M1-like antitumoral phenotype, and 4) TAMs-mediated delivery of therapeutic cargoes [10, 24, 25].

#### Blocking monocytes/macrophages recruitment

One strategy for targeting TAMs is to block their recruitment or infiltration of monocytes/macrophages into tumors. TAMs replenishment in the tumor is often mediated by monocytic recruitment via the CCL-2-C-C chemokine receptor (CCR)-2 axis [12, 59]. CCL-2 released by tumor cells, macrophages, and stromal cells within the TME recruits monocytes that express the receptor CCR-2 and granulocytes (i.e. myeloid-derived suppressor cells; MDSCs) that express the receptor CCR-5 to tumors, thereby driving tumor progression. Thus, targeting the CCL-2-CCR-2 axis can reduce numbers of TAMs in the tumor milieu [12, 59]. Combination therapy of CCL-2 or CCR-2 blockade with chemotherapy, radiation therapy or immunotherapy reduces the infiltration of myeloid cells and results in improved antitumor effects in preclinical models [60, 61]. Clinical trials with several CCR-2 inhibitors (PF-04136309, MLN1202, BMS-813160, and CCX872-B) are currently ongoing for the treatment of solid tumors [24]. CCL-2 inhibition reduces tumor growth and metastasis in preclinical models, when administered in combination with chemotherapy, neutralization of CCL-2 improved the efficacy of treatment. However, it has also been observed that cessation of anti-CCL-2 treatment leads to a rebound effect, with increased release of the monocytes previously trapped within the bone marrow, thus accelerating breast cancer metastasis by promoting angiogenesis [62]. Nevertheless, a CCL-2-blocking antibody, carlumab (CNTO 888), and a CCR-2 small molecule inhibitor (PF-04136309) are the main drugs currently being tested [10, 11, 25]. PF-04136309 has shown some benefit in pancreatic cancer patients in combination with the FOLFIRINOX (a chemotherapeutic regimen of folinic acid, fluorouracil, irinotecan, and oxaliplatin for pancreatic cancer patients). Patients treated with PF-04136309 plus FOLFIRINOX did not experience worse toxicity than those receiving chemotherapy alone. Patients treated only with FOLFIRINOX did not show an objective response. By contrast, in combination group, 16 of 33 imaging-evaluated patients had an objective tumor response (49%), and 32 of those patients achieved local tumor control (97%) [60].

Other pathways also involved in TAM recruitment include the C-X-C ligand (CXCL)-12-C-X-C receptor (CXCR)-4 axis and VEGF receptor pathway. Stromal cell-derived CXCL-12 is another chemokine that facilitates the migration of macrophages through endothelial barriers and drives TAM accumulation and survival in hypoxic areas of tumors [63]. Thus, blocking CXCL-12-CXCR-4 signaling represents a promising strategy to modulate macrophage infiltration and prevent metastasis [64–66]. Indeed, targeting CXCR-4 in several preclinical models, including breast, prostate, and ovarian cancer, was shown to significantly reduce total tumor burden and metastases [65, 66]. In addition, blockade of CXCL-12/CXCR-4 axis prevented the post-sepsis-induced tumor progression, TAM accumulation, and TAM in situ proliferation [64]. Vascular endothelial growth factor (VEGF) also functions to recruit macrophages into tumors, which requires VEGFR2 expressed by the macrophages [67]. Selective inhibition of VEGFR2 results in reduced macrophage infiltration and decreased angiogenesis in breast and pancreatic cancer models.

Colony-stimulating factor 1 (CSF1) controls proliferation, differentiation, recruitment, survival, and function of mononuclear phagocytes (e.g., macrophages, monocytes). CSF1 receptor (CSF1R) signaling in TAMs may promote their acquisition of an immunosuppressive and pro-tumorigenic, M2-like phenotype [68, 69]. Thus, the CSF1/CSF1R signaling axis is an obvious and attractive target. CSF1R belongs to the tyrosine kinase transmembrane receptor family and binding to its ligand (e.g., CSF1, IL34) induces homodimerization of the receptor and subsequent activation of receptor signaling [68]. Based on the development of a number of small-molecule and antibody antagonists to prevent receptor dimerization, current research is thereby focused on abrogating binding to CSF1 and activation of signaling, decreasing TAM infiltration and augmenting the effect of antitumor immune in the tumor [70, 71]. Macrophage recruitment can be remarkably reduced by blocking the CSF1/CSF1R axis, which has recently been reviewed elsewhere [11, 24, 25, 68]. Based on these preclinical data, several clinical trials of CSF1/CSF1R inhibitors have been completed or are ongoing. Among the small molecules, PLX3397, a small-molecule CSF1R inhibitor that can be administered orally, has the broadest clinical development, as it is being tested clinically in a variety of malignancies [72, 73]. Blockade of CSF1R with PLX3397 induced clinical regression in patients and decreased the intratumoral accumulation of immunosuppressive macrophages, thus confirming the validity of TAMs as a therapeutic target in cancer therapy [70, 71]. Several other small molecules are also being tested clinically including PLX7486, BLZ945, JNJ-40346527, and ARRY-382. Another strategy involves monoclonal antibodies designed to target either CSF1R (AMG820 and IMC-CS4) or its ligand CSF1 (MCS110 and PD-0360324) (also reviewed in [11]). The preclinical data strongly suggest that targeting the CSF1/CSF1R axis has the potential to complement conventional therapeutic strategies. While targeting monocyte/macrophage recruitment before their arrival to the tumors is effective in various preclinical cancer models, TAMs can also be directly targeted by other strategies once they invade the tumors. Strikingly, blockade of CSF1R also induces an extensive metabolic rewiring that culminates with the restoration of glycolysis to favor the maintenance of M1-like TAMs [74]. Thus, TAMs metabolism and the immunometabolic circuitries linking TAMs to other stromal cells within the TME may also be harnessed in combination with immunotherapeutic agents for cancer immunotherapy, which has recently been reviewed [75].

#### Direct TAMs depletion

The selective elimination of TAMs in tumors has been explored for cancer therapy. One attractive strategy for depleting TAMs within the tumor milieu is to trigger their apoptosis, which could effectively inhibit tumor growth and restore local immune surveillance in the TME. Mechanistically, in addition to preventing TAMs recruitment into the tumors, blockade of the CSF1/CSF1R axis has been shown to reduce macrophage survival [70, 71]. Several compounds have been shown to induce apoptosis of macrophages including zoledronate, clodronate, and trabectedin as will be reviewed as the following.

Bisphosphonates, a class of anti-resorptive drugs, are taken up by phagocytosing cells and have cytotoxic effects on myeloid cells [76]. Based on their structure, bisphosphonates could be divided into two categories, non-nitrogen-containing and nitrogen-containing categories [76]. Bisphosphonates have traditionally been used in the clinic to prevent or inhibit the development of bone metastases or excessive bone resorption and for therapy of inflammatory diseases [77]. TAMs are a potential target of the bisphosphonates, which has been reviewed [78]. Moreover, bisphosphonates exert a range of direct and indirect anti-tumor effects, including inhibition of tumor cell proliferation and induction of tumor cell apoptosis, inhibition of tumor cell adhesion and invasion, anti-angiogenesis, synergism with anti-neoplastic drugs, and enhancement of immune surveillance (reviewed in [79]). Clodronate, currently used in the treatment of osteoporosis and bone metastasis, belongs to the drug family of bisphosphonates. Liposomes with encapsulation of clodronate act as an efficient reagent for selective depletion of macrophages. Indeed, these clodronate-liposomes, due to their big size, are rapidly recognized and engulfed by macrophages, resulting in the apoptosis of the host cells [80]. Notably, large, multilamellar liposomes containing clodronate have been developed and successfully applied in several cancer models, leading to the regression of tumor growth, angiogenesis and metastasis [81]. The benefits of macrophage depletion is not only seen with clodronate, but also with other bisphosphonates, of which zoledronate has been shown to be the most active [77]. Zoledronate is a third-generation nitrogen-containing bisphosphonate that has been shown to exhibit selective cytotoxicity to matrix metalloproteinase-9 (MMP9)-expressing TAMs, and to impair differentiation of myeloid cells into TAMs, which improves tumoricidal activity of macrophages [82].

Trabectedin (ET-743, Yondelis®) is a tetrahydroisoquinoline alkylating agent, originally extracted from a marine organism, the turbinate Ecteinascidia, but can now be produced synthetically [83, 84]. It has been approved in Europe and other countries as a second-line therapy for treating patients with advanced soft tissue sarcoma after failure of doxorubicin or ifosfamide and in relapsed platinum-sensitive ovarian cancers [85]. It binds the minor groove of DNA and can cause cancer cell death by inducing cell cycle arrest, blocking active transcription and inflicting DNA double-strand breaks [86]. Intriguingly, trabectedin specifically targets mononuclear phagocytes, including TAMs, by activation of the caspase 8 cascade via TNF-related apoptosis-inducing ligand (TRAIL) receptors [87]. Unlike other leukocyte subsets, monocytes that express very low levels of TRAIL decoy receptors are exquisitely sensitive to TRAIL [88]. Patients treated with trabectedin have reduced TAMs density correlated with a reduction in angiogenesis [87]. Taken together, depleting TAMs by stimulating their apoptosis may be of high therapeutic potential in limiting tumor development. Thus, macrophage destruction within the tumor is being further explored in the setting of preclinical models and clinical trials in different cancers.

Although depletion of TAMs could delay tumor progression, whether only immunosuppressive myeloid cells are targeted remain unclear. Likely, immunoprotective cells will also be depleted, causing severe adverse effects, such as bacterial infections. Hence, complete deletion approaches for myeloid cells are not feasible in the context of cancer.

#### Re-educating TAMs

In addition to directly depleting TAMs, it is also appealing to revert the activated state of the pro-tumorigenic TAMs into a quiescent state or even to induce them to acquire tumor-suppressive phenotypes. As discussed above, macrophages are functionally plastic, as the cells may respond differently to an alternation of molecules in the TME, including chemokines, cytokines, pattern recognition receptors and hormones [8]. Thus, to switch tumor-promoting M2-like TAMs to a tumoricidal M1-like phenotype by manipulating environmental stimuli is a potential strategy for tumor eradication. Tellingly, macrophages have been shown to be required for the efficacy of chemotherapy and immunotherapy [89, 90], which undermines the rational for depleting TAMs during cancer therapy. Alternatively, there are multiple strategies of reorienting rather than directly depleting TAMs for cancer therapy, which has recently been reviewed elsewhere [10, 11, 24], including targeting both the tumor cells (e.g., CD47 antibody) and the TAMs (e.g., CSF1/CSF1R blockades, TLR agonists, PI3Kγ inhibitors, CD40 agonists, and Class IIa histone deacetylase (HDAC) inhibitors). Hence, we will briefly review studies related to this strategy here (also described in Fig. 2).

TAMs express surface receptors that bind the Fc fragment of antibodies and enable them to engage in antibody-dependent cellular cytotoxicity/phagocytosis (ADCC/ADCP), which are amenable to therapeutic strategies capitalizing on the effector function of TAMs [10]. Signal regulatory protein alpha (SIRPα) is an ITIM-bearing inhibitory receptor expressed on myeloid cells, including macrophages. It recognizes CD47, which acts physiologically as a “don’t eat me” signal and is found to be ubiquitously overexpressed on many different types of cancer. Interactions between CD47 and SIRPα prevent tumor cells from undergoing phagocytosis, allowing cancer cells to escape immune surveillance [91]. Masking of CD47 on tumor cells using monoclonal antibody or soluble SIRPα-Fc construct can trigger ADCP of tumor cells by TAMs [92, 93]. Enabling phagocytosis by macrophages in the tumor can lead to induction of effective immune responses against cancer [94]. However, enhancement of macrophage-dependent ADCP via interference with the inhibitory CD47-SIRPα pathway might involve mechanisms that lie beyond pure activation of TAMs effector function. ADCP elicited by targeting the CD47-SIRPα axis resulted in functional skewing of mouse macrophages towards an M1-like phenotype in tumor models, thus contributing to antitumor immune responses [95]. Of note, multiple therapies targeting the CD47-SIRPα axis are under preclinical and clinical investigation, including conventional antibodies, recombinant polypeptides, and bispecific molecules [96]. It should be noted, however, that engagement of macrophage-Fcγ receptors by therapeutic antibodies was shown to enhance the immunosuppressive, proangiogenic, and protumoral functions of TAMs [97]. Agents that enhance TAMs-mediated ADCP should potentially be used in combination with checkpoint blockade therapy [98], which could stimulate effective antitumor immunity.

CD40, a member of the TNF receptor family, is broadly expressed on many cell types, including APCs, DCs, B cells, macrophages, monocytes, as well as a number of nonhematopoietic cell types and some tumor cells [99]. When CD40 signaling is initiated and activated on APCs by the engagement of the ligand of CD40 (CD40L) expressed mainly on activated T helper cells, APCs release proinflammatory cytokines, and increase costimulatory molecules, such as CD80 and CD86, which may help support antitumor T cell activity [99]. To date, CD40 agonist antibodies show combinatorial efficacy in pancreatic cancer with gemcitabine, resulting in the regression of tumors by promoting antitumor macrophages that acquired antigen-presenting capabilities, re-establishing immune surveillance as well as prolonging patient survival [100, 101]. Clinical trials combining CD40 agonists and recombinant CD40Ls with chemotherapy, immunotherapy, vaccines, and angiogenic inhibitors are currently ongoing. In mouse models of cancer, CSF1R blockades synergize with the agonistic CD40 antibodies to remove inhibitory immune populations and to drive endogenous antitumor immune responses, resulting in improved tumor clearance and significantly lengthened overall survival [102]. Intriguingly, one study has suggested that TAMs depletion using CSF1R blockade diminished the antitumor activity of infused effector CD8^+^ T cells, whereas TAMs programming with agonistic CD40 antibody enhanced the accumulation and longevity of TCR-engineered cells [103]. Echoing the above observations, reprogramming TAMs to anti-tumor phenotype, rather than targeted ablation of the TAMs, may be the preferable therapeutic paradigm for cancer therapy.

Other strategies to activate TAMs for antitumor therapy include the targeting of phosphoinositide 3-kinase (PI3K)-γ, which is one of the four Class I PI3K p110 catalytic isoforms and is also activated in TAMs downstream of multiple pathways. Activation of PI3Kγ signaling selectively drives immunosuppressive transcriptional programing in macrophages that inhibits adaptive immune response and promotes myeloid cells invasion into tumors [104]. In mouse tumor models, pharmacological inhibition of PI3Kγ using IPI-549, a PI3Kγ-selective inhibitor, results in macrophage reprogramming, which reduced protumor macrophages while increasing antitumor macrophages and T cell responses [105]. Additionally, epigenetic reprogramming of macrophages via inhibition of histone deacetylases (HDACs), enzymes that regulate activity of many transcription factors, can also elicit T cell responses [106]. In mammary tumor models, TMP195, a selective Class IIa HDAC inhibitor, enabled TAMs populations within tumors to acquire antitumor phenotypes that support T cell responses [106].

The macrophage receptor with collagenous structure (MARCO), a member of the class A scavenger receptor family, is a nonopsonic phagocytic receptor mainly expressed by macrophages, dendritic cells and certain endothelial cells, wherein it is appreciated for its role in sensing and clearing pathogens though the recognition of pathogen-associated molecular patterns (PAMPs) [107]. Strikingly, mounting evidence has recently indicated that MARCO also plays important roles in regulating macrophage polarization [108, 109]. For instance, a study conducted on alveolar macrophages demonstrated that MARCO acts as an initial signaling receptor for asbestos and polarizes macrophages to a profibrotic M2 phenotype [108]. Additionally, MARCO is also expressed by a subpopulation of TAMs with an M2-like immunosuppressive gene signature in the TME of both murine tumor models and in human cancer. Treatment with antibodies targeting MARCO resulted in reduced tumor growth and inhibition of metastasis and a switch to a more proinflammatory macrophage phenotype [108]. Of note, MARCO is also a viable target for chimeric antigen receptor (CAR) T cell strategies for cancer therapy which has emerged as an important immunotherapeutic approach. CAR T cell therapy is a revolutionary milestone for the treatment hematological malignancies (such as B cell acute lymphoblastic leukemia) [110–112]. However, its efficacy in the treatment of solid tumors still needs to be further improved. As MARCO supports proinflammatory signals, CAR T cells targeting MARCO have the potential to reinforce other immunotherapeutic modalities, including checkpoint blockade-based immunotherapy. Thus, further studies are needed to determine whether co-expression of two different CARs, one for TAMs and another for tumor cells, can enhance therapeutic effects of CAR T cell transfer therapy. All of these studies suggest that future cancer therapy should focus not only on cancer cells but also on TAMs by their reorientation or by selective depletion in the TME.

Complement is a central part of the innate immune system that acts as a first defense against pathogens and triggers release of inflammatory cytokines [113]. The physiological functions of complement are traditionally believed to defend against microbes and unwanted host molecules, to bridge innate and adaptive immunity, and to bind to immune complexes for complement-mediated lysis [114]. However, complement activation is also involved in many more inflammatory and immunological processes, including those that occur in the tumor context. Strikingly, complement has been implicated in the elimination of tumor cells by complement-fixing antibodies, paradoxically, complement-induced inflammation can also promote tumor progression [115]. The protumoral effect of the complement system is achieved through establishing an immunosuppressive microenvironment, promoting angiogenesis, modulating immune cells, and enhancing tumor cell proliferation, invasion and migration [115, 116]. Complement effectors such as C1q, anaphylatoxins C3a and C5a inhibit the antitumor immune response through the recruitment and/or activation of immunosuppressive cell subpopulations, including MDSCs, Tregs, or TAMs [115]. Macrophages express cognate receptors for both C3a and C5a on their cell surface, and specific binding of C3a and C5a affects the functional modulation and alters the TME and tumor immunity. Thus, the complement system has emerged as a target for cancer immunotherapy. In mouse model of squamous carcinogenesis, urokinase (uPA)^+^ macrophages release C5a in a C3-independent manner during premalignant progression. C5a regulates pro-tumorigenic properties of C5aR1^+^ mast cells and macrophages, resulting in immunosuppressive macrophage polarization and inhibition of CD8^+^ T cell activation [117]. While neither PMX-53 (a C5aR1 peptide antagonist) nor paclitaxel alone significantly altered tumor progression, a combination of the two synergized to effectively inhibit tumor growth by repolarizing TAMs towards the M1-like phenotype that not only affects angiogenic programs but also leads to recruitment of cytotoxic T lymphocytes [117]. Another study has also demonstrated that intracellular activation of complement C3 derived from tumor cells suppressed the infiltration and function of CD8^+^ cytotoxic T lymphocytes by promoting the accumulation and immunosuppressive activity of TAMs in a C3aR-dependent, rather than C5aR-dependent manner, resulting in activation of PI3Kγ signaling that mediates TAMs immunosuppressive activity [118]. Strikingly, blocking tumor-derived complement C3 is sufficient to enhance antitumor immunity [118]. Taken together, the complement pathway in the tumor setting might be potentially exploited as a target to reeducate TAMs for cancer therapy, but given the complex interactions of complement system in the tumor context, such therapy strategies should be developed carefully and with clearly outlined biological endpoints. Attempts to revert tumor-promoting TAMs or to alter their phenotype could provide new opportunities for the development of novel antitumor therapies.

#### TAMs-mediated delivery of therapeutics

A new generation of drug delivery methods, so called live cell-mediated drug delivery systems, consists in the use of the host cells (i.e. monocytes [119], macrophages [120], mesenchymal stem cells [121]), either as a whole or by employing selected key components of these cells, as ‘Trojan Horses’ loaded with drugs [119, 122]. Macrophages, professional phagocytic cells, are non-immunogenic, which endows them long blood-circulation times, and high phagocytic capability that enables them to extensively internalize and hold considerable drug loadings, both of which are prerequisites for drug delivery carriers [8, 22]. Moreover, macrophages are one of the most abundant types of circulating cells, which can be easily separated, loaded with drugs ex vivo, and reintroduced into the circulation. Of most importance, macrophages represent most of the leukocytes in tumors and account for up to 50% of cells in a tumor mass [8, 22]. Thus, the use of natural living macrophages as drug delivery carriers may be a potential strategy for cancer therapy.

Macrophages receive considerable interest as a drug delivery carrier due to their tropism to hypoxia and their ability to migrate and infiltrate into tumors. It is crucial that macrophages are alive in circulation to ensure their tumor-homing capacity and to unload the drug upon their arrival at tumor sites [123]. However, most antitumor drugs cannot be directly loaded into macrophages because their high cytotoxicity will quickly kill the cells. Alternatively, loading nanoparticles into macrophages may be a feasible approach. Inspired by the uptake of antitumor nanoparticles by macrophages, researchers have employed nanoparticles that can be taken by TAMs to combat cancer [124, 125]. Macrophage-mediated drug delivery systems designed with precisely controlled drug release when macrophages reach the target sites would minimize adverse effects of the loaded drug on the macrophages to reserve their functions, and maximize the drug loading content. Therefore, it is desirable to develop systems that take the advantages of both nanomedicine and macrophages in tumor-targeting [124]. Up to date, relatively few studies have assessed the in vivo antitumor efficacies of macrophage-mediated drug delivery systems. Choi et al. showed improved in vivo tumor accumulation of liposomal doxorubicin (Dox) when delivered by macrophages, exhibiting higher therapeutic efficacy in a subcutaneous tumor model than liposomal-Dox or Dox alone [120]. Because premature drug release in macrophages before their arrival at the target site will cause cell death or dysfunction, the precise control of the drug release in macrophage-mediated drug delivery system should be considered. A silica-based drug nanocapsule, consisting of a drug-silica complex filling and a solid silica sheath, may solve this issue [126]. When taken up by macrophages, the silica-based drug nanocapsule minimally release drug molecules in the early hours of cell entry, allowing macrophages to home to tumors and release drugs within tumors. Therapy with Dox-laden nanocapsule leads to efficient tumor growth suppression, while causing little systemic toxicity [126].

Although macrophages may truly carry “active” nanomedicines towards tumors, the filed is still in its infancy and many challenges remain. Of most importance, since macrophages may exhibit both protumoral and antitumoral activities in TME, it is important to fully understand the in vivo fate and functional state of these macrophages and ideally, to promote and maintain the anti-tumor functional state of the carrier macrophages to further maximize this cell-directed therapy. Further development of the technology for delivery of biologics, such as peptides or drugs that may enhance in vivo antitumor properties of these macrophages would greatly expand the utility of macrophage-mediated drug delivery system in cancer therapy. Finally, other selective drug delivery strategies using macrophages await further investigation. For instance, macrophages have been used to facilitate virus infection by providing a cell carrier for oncolytic virus delivery [127, 128]. Macrophage-based virotherapy was shown to mount efficient attack on tumors where the virus could infect tumor cells, achieving significant antitumor efficacy.

### Targeting TAMs potentiates the efficacy of checkpoint blockades

Over the last decade, the focus of cancer treatment has shifted from the tumor to the host. The goal of cancer immunotherapy is to harness or mobilize the immune system, both innate and adaptive, to attack and destroy tumors in cancer patients [1]. However, cancer could effectively suppress antitumor immune responses by activating negative regulatory pathways (also known as checkpoints) that are associated with immune homeostasis or by adopting features that enable them to actively escape detection [2]. Checkpoint blockade-based immunotherapy that unleashes antitumor immune reactions has resulted in unprecedented rates of durable responses in a large set of cancers. Checkpoint inhibition eliminates the brakes that hamper the effective recognition and elimination of tumor cells by immune cells. To date, two such immune checkpoints, CTLA4 and PD-1, have garnered the most attention [129]. Although such treatments often yield sustained benefits, many patients do not respond or develop resistance, and complete cures with single immunotherapy agents only occur in a minority of patients [2, 130, 131]. Ongoing experimental, preclinical and clinical studies indicate that both tumor cell-extrinsic and tumor cell-intrinsic factor contribute to the resistance to current immunotherapies [130, 131]. As discussed above, TAMs are a major component of solid tumors and influence various aspects of cancer progression. Moreover, TAMs can also express PD-L1 and PD-L2, as well as CD80 and CD86, and the related protein B7-H4, which contribute to the establishment of the immunosuppressive TME [8, 11, 25]. Not surprisingly, rescuing TAM-mediated immune dysfunctions by ablation or repolarization demonstrate combinatorial efficacy when combined with immunotherapy, especially with checkpoint blockade that aim to reverse the immunosuppressive nature of the TME and restore cytotoxic T lymphocytes against tumor cells. To date, many clinical trials examining these combinations are in progress (also see Table 1).

**Table 1:** Combination TAMs-directed therapies with checkpoint blockades in selected clinical trials for cancer therapy

Action	TAM-targeted agent	Checkpoint blockade	Clinical phase (status)	Tumor type	Effect	Clinical trials
Targeting TAM recruitment and survival	Emactuzumab (CSF1R inhibitor)	Atezolizumab (PD-L1 antibody)	Phase I (Recruiting)	Locally advanced or metastatic solid tumors	NA	NCT02323191
	ARRY-382 (CSF1R antagonists)	Pembrolizumab (PD-1 antibody)	Phase I/II (Active, not recruiting)	Advanced solid tumors	NA	NCT02880371
	Pexidartinib (anti-CSF1R)	Durvalumab (PD-L1 antibody)	Phase I (Active, not recruiting)	Colorectal cancer; Pancreatic cancer; Metastatic cancer; Advanced cancer	NA	NCT02777710
	SNFX-6352 (CSF1R antagonists)	Durvalumab (PD-L1 antibody)	Phase I (Recruiting)	Solid tumor; Metastatic tumor; Locally advanced malignant neoplasm; Unresectable malignant neoplasm	Safe, Well-tolerated	NCT03238027
	BLZ945 (CSF1R antagonists)	PDR001 (anti-PD-1; Novartis)	Phase I/II (Recruiting)	Advanced solid tumors	NA	NCT02829723
	Cabiralizumab (CSF1R antagonists)	Nivolumab (anti-PD-1)	Phase I (Recruiting)	Advanced malignancies	Safe, Well-tolerated	NCT03158272
	AMG820 (CSF1R antagonists)	Pembrolizumab (PD-1 antibody)	Phase I/II (Active, not recruiting)	Pancreatic cancer; Colorectal cancer; Non-small cell lung cancer	NA	NCT02713529
	Trabectedin	Durvalumab (PD-L1 antibody)	Phase I (Recruiting)	Ovarian carcinoma; Soft tissue sarcoma	NA	NCT03085225
Reprogramming TAMs to antitumor macrophages	APX005M (CD40 agonistic antibody)	Nivolumab (anti-PD-1)	Phase I/II (Recruiting)	Non-small cell lung cancer; Metastatic melanoma	NA	NCT03123783
	Selicrelumab (CD40 agonist)	Atezolizumab (PD-L1 antibody)	Phase I (Recruiting)	Solid tumors	NA	NCT02304393
	IPI-549 (PI3Kγ inhibitors)	Nivolumab (anti-PD-1)	Phase I (Recruiting)	Advanced solid tumor; non-small cell lung cancer; melanoma; breast cancer	NA	NCT02637531
	TTI-621 (SIRPα-IgG1 Fc)	Nivolumab (anti-PD-1); Pembrolizumab (PD-1 antibody); Atezolizumab (PD-L1 antibody); Durvalumab (PD-L1 antibody)	Phase I (Recruiting)	Solid tumors; melanoma; merkel-cell carcinoma; squamous cell carcinoma; breast carcinoma	NA	NCT02890368
	TTI-621 (SIRPα-IgG4 Fc)	Nivolumab (anti-PD-1); Pembrolizumab (PD-1 antibody);	Phase I (Recruiting)	Lymphoma; myeloma	NA	NCT03530683
	GSK3145095 (RIP inhibitor)	Pembrolizumab (PD-1 antibody)	Phase I/II (Recruiting)	Neoplasms; pancreatic	NA	NCT03681951
	NKTR262 (TLR7/8 agonist)	Nivolumab (anti-PD-1)	Phase I/II (Recruiting)	Melanoma; merkel cell carcinoma; breast cancer; renal cell carcinoma; colorectal cancer	NA	NCT03435640

*NA* Not available, *CSF1R* Colony-stimulating factor 1 receptor, *SIRPα* Signal regulatory protein alpha, *RIP1* Receptor-interacting serine/threonine protein kinase 1, *TLRs* Toll-like receptors

TAMs are a key component of the immunosuppressive pathway targeted by the blockade of immune checkpoints. As mentioned above, several TAM-directed targeting strategies are undertaken to decrease the number of suppressive macrophages within tumors, which can be leveraged to increase the efficacy of immune checkpoint blockade. Accordingly, CSF1/CSF1R blockade could improve the efficacy of a diversity of immunotherapeutic modalities, including PD-(L)1 or CTLA-4 blockades. For instance, treatment with CSF1R antagonists in combination with checkpoint blockade-based immunotherapy in the mouse models of pancreatic, breast, cervical, and ovarian cancer results in delaying tumor progression [10, 24, 68, 69]. These studies provide a proof of concept that targeting TAMs could boost the efficacy of checkpoint blockade-based immunotherapy, leading to a number of clinical trials combining CSF1 and/or CSF1R inhibitors with the blockade of immune checkpoints. In a promising study in patients with pancreatic cancer, which does not traditionally respond to immunotherapy, when CSF1R antagonists and PD-1 blockade were combined, responses in some patients were observed, and these studies are now moving forward to a multi-arm phase II clinical trial (reviewed in [25]). These results indicate that the TAM depletion by targeting CSF1R can improve the efficacy of checkpoint inhibitors.

In addition, reprogramming of TAMs can also enhance the antitumor effects of checkpoint inhibitors. For instance, TMP195 could repolarize TAMs to M1-like phenotype and to synergize with PD-1 antibody to reduce tumor burden and metastasis in an autochthonous mouse model of breast cancer [106]. Similarly, administration of neutralizing antibody against MARCO enhances the efficacy of anti-CTLA-4 antibody treatment in mice with melanoma [108]. Furthermore, PI3K inhibition markedly enhances the tumor suppressive effects of checkpoint inhibition of PD-1 in multiple mouse tumor models [105, 132, 133]. Receptor-interacting serine/threonine protein kinase 1 (RIP1) is upregulated in both human and mouse TAMs in pancreatic ductal adenocarcinoma (PDA). Targeting RIP1 led to the reprogramming of TAMs toward an M1-like phenotype and tumor suppression. Moreover, RIP1 inhibition synergizes with PD-1- and inducible co-stimulator-based immunotherapies to suppress tumor growth in mouse models of PDA [134]. Clinical trials are currently underway to test the combination of the RIP1 inhibitor GSK3145095 and pembrolizumab in adults with advanced solid tumors (NCT03681951). Another target for macrophage repolarization is Toll-like receptors (TLRs) that stimulate innate immune response. TLR agonists comprise alternative strategies to elicit antitumor immune responses that have been developed for cancer therapy [135]. For example, local delivery of a TLR7/8 agonist 3 M-052 boosted systemic antitumor immunity by repolarizing TAMs to M1-like phenotypes and resulted in tumor regression in a mouse model of subcutaneous melanoma [136]. Combining 3 M-052 with antibodies against CTLA-4 and PD-L1 was synergistic in inhibiting tumor growth [136]. Though clinical evidence indicating the efficacy of TLR agonists is still insufficient, multiple clinical trials are underway. For instance, NKTR-262, another TLR7/8 agonist, is currently under evaluation for the treatment of melanoma and other advanced cancers in combination with the checkpoint inhibitor nivolumab (NCT03435640).

Taken together, TAMs contribute to the immunosuppression observed in TME via multiple mechanisms, thus, targeting of TAMs could complement immune checkpoint blockades by removing other negative factors that might continue to restrain the action of T cells despite checkpoint blockade. Although therapeutic effects of the combining checkpoint blockade with TAM intervention are evident from the previous pre-clinical studies, further basic researches will be required to apply this novel strategy to the clinic arena.

### Conclusions, challenges and perspectives

Given the important roles of TAMs in orchestrating tumor progression, targeting TAMs offers a novel approach to improving antitumor therapy. Various therapeutic strategies have been developed with TAMs or their functional mediators as direct targets, including TAMs depletion, blockade of monocytes/macrophage recruitment, and the reprogramming TAMs into proinflammatory M1-like macrophages or neutralizing the products of TAMs. Although most TAMs-targeting strategies are still at the preclinical stage, several antagonists that can be used for TAMs depletion have already been tested in clinical trials for solid tumors. Further investigation of synergistic effects of targeting TAMs with checkpoint blockade-based immunotherapies will lead to the improvement of ongoing immunotherapeutic strategies.

To expedite the leap from bench to bedside, several challenges and unmet needs for TAMs-targeted therapies must be overcome. Many questions remain to be answered. Does choice of approaches, direct depletion or reprogramming of TAMs, depend on tumor type? Do the currently targeted signaling pathways mechanistically overlap or synergize in vivo? What are the long-term consequences of repolarizing macrophages towards a proinflammatory state? More importantly, regarding phenotypic reversibility, which macrophage transcription factors are critical for promoting tumor immunosuppression and immune activation? Which TAM subsets play a role in promoting tumorigenesis and which subtypes should be targeted for anti-tumor therapy? How do epigenetic factors govern the unique gene expression patterns and biological behavior of TAMs, and are they stably inherited? Answering these questions will unleash the potential of TAMs-targeted therapies as novel antitumor strategies.

The functional significance of TAMs in TME makes them attractive targets for cancer therapy, however, because there are complex intercellular interactions involving TAMs in the TME, targeting TAMs may trigger multifaceted stromal reactions in the tumor milieu that are difficult to predict and may vary from patient to patient. Although the details of network of TME remain unclear, various strategies could be used to modify the tumor milieu, including remodeling tumor vasculature, removing immunosuppressive cells, mobilizing immune effector cells to kill tumors, and reprogramming stroma to enhance the delivery of antitumor agents. As such, targeting TAMs could not only inhibit the tumor “seeds” but also renovate the tumor “soil” to construct a tumor-suppressive microenvironment, thereby turning foes that promote tumor progression into friends that suppress tumor development. TAM-targeting strategies can per se result in therapeutic benefits. However, to ultimately eradicate tumor, our tenet is that synergistic combinations of TAMs-directed therapeutics and other effective treatments such as immunotherapy should also be considered.

Currently, TAMs-targeted therapeutics are rapidly being explored and developed. The extensive use of single-cell sequencing, multiplex immunohistochemistry and mass cytometry techniques will considerably enhance our knowledge on the heterogeneity of TAMs in tumor milieu. Selective elimination of the tumor-promoting TAMs subsets or repurposing them as tumor-sabotaging elements could become an effective therapeutic approach utilized alone or in combination with other therapeutic strategies for cancer therapy.

## Data Availability

The material supporting the conclusion of this review has been included within the article.

## Abbreviations

ADCC: Antibody-dependent cellular cytotoxicity
ADCP: Antibody-dependent cellular phagocytosis
APCs: Antigen-presenting cells
ARG1: Arginase 1
CAR: Chimeric antigen receptor
CCL: C-C chemokine ligand
CCR: C-C chemokine receptor
CSF1: Colony-stimulating factor 1
CTL: Cytotoxic T lymphocytes
CTLA-4: Cytotoxic T-lymphocyte antigen 4
CXCL: C-X-C ligand
CXCR: C-X-C receptor
DCs: Dendritic cells
FOXP3: Forkhead box P3
HDACs: Histone deacetylases
HLA: Human leukocyte antigen
IFN: Interferon
IL: Interleukin
iNOS: Inducible nitric oxide synthase
LIR1: Leukocyte immunoglobulin-like receptor subfamily B member 1
LPS: Lipopolysaccharide
MARCO: Macrophage receptor with collagenous structure
MDSCs: Myeloid-derived suppressor cells
MHC: Major histocompatibility complex
MMP9: Matrix metalloproteinase-9
PAMPs: Pathogen-associated molecular patterns
PD-1: Programmed cell death protein 1
PGE2: Prostaglandin-E2
PI3K: Phosphoinositide 3-kinase
RIP1: Receptor-interacting serine/threonine protein kinase 1
SIRPα: Signal regulatory protein alpha
TAMs: Tumor-associated macrophages
TCR: T cell receptor
TGF-β: Transforming growth factor-β
TLRs: Toll-like receptors
TME: Tumor microenvironment
TRAIL: TNF-related apoptosis-inducing ligand
Tregs: Regulatory T cells
VEGF: Vascular endothelial growth factor
VISTA: V-domain Ig-containing suppressor of T cell activation
